# Implementation of safety checklists in surgery: a realist synthesis of evidence

**DOI:** 10.1186/s13012-015-0319-9

**Published:** 2015-09-28

**Authors:** Brigid M. Gillespie, Andrea Marshall

**Affiliations:** NHMRC Centre for Research Excellence in Nursing (NCREN), Centre for Health Practice Innovation (HPI), Menzies Health Institute Qld (MHIQ), Griffith University, Gold Coast Campus, Gold Coast, QLD 4222 Australia; School of Nursing and Midwifery, Griffith University, Gold Coast Campus, Gold Coast, QLD 4222 Australia; Gold Coast University Hospital, Gold Coast Hospital and Health Service, Southport, QLD 4215 Australia

## Abstract

**Aim:**

The aim of this review is to present a realist synthesis of the evidence of implementation interventions to improve adherence to the use of safety checklists in surgery.

**Background:**

Surgical safety checklists have been shown to improve teamwork and patient safety in the operating room. Yet, despite the benefits associated with their use, universal implementation of and compliance with these checklists has been inconsistent.

**Data sources:**

An overview of the literature from 2008 is examined in relation to checklist implementation, compliance, and sustainability.

**Review methods:**

Pawson’s and Rycroft-Malone’s realist synthesis methodology was used to explain the interaction between context, mechanism, and outcome. This approach incorporated the following: defining the scope of the review, searching and appraising the evidence, extracting and synthesising the findings, and disseminating, implementing, and evaluating the evidence. We identified two theories a priori that explained contextual nuances associated with implementation and evaluation of checklists in surgery: the Normalisation Process Theory and Responsive Regulation Theory.

**Results:**

We identified four a priori propositions: (1) Checklist protocols that are prospectively tailored to the context are more likely to be used and sustained in practice, (2) Fidelity and sustainability is increased when checklist protocols can be seamlessly integrated into daily professional practice, (3) Routine embedding of checklist protocols in practice is influenced by factors that promote or inhibit clinicians’ participation, and (4) Regulation reinforcement mechanisms that are more contextually responsive should lead to greater compliance in using checklist protocols. The final explanatory model suggests that the sustained use of surgical checklists is discipline-specific and is more likely to occur when medical staff are actively engaged and leading the process of implementation. Involving clinicians in tailoring the checklist to better fit their context of practice and giving them the opportunity to reflect and evaluate the implementation intervention enables greater participation and ownership of the process.

**Conclusions:**

A major limitation in the surgical checklist literature is the lack of robust descriptions of intervention methods and implementation strategies. Despite this, two consequential findings have emerged through this realist synthesis: First, the sustained use of surgical checklists is discipline-specific and is more successful when physicians are actively engaged and leading implementation. Second, involving clinicians in tailoring the checklist to their context and encouraging them to reflect on and evaluate the implementation process enables greater participation and ownership.

**Electronic supplementary material:**

The online version of this article (doi:10.1186/s13012-015-0319-9) contains supplementary material, which is available to authorized users.

## Introduction

Approximately 40 % of adverse events (AE) occur in the operating room (OR), and up to 50 % of these are considered avoidable errors [1]. In industrialised countries, mortality rates associated with surgery are estimated to be 0.4 to 0.8 % [2] but may be as much as 10 times higher in developing countries [3]. In response to the need to minimise the potential of AE in high-risk clinical environments, patient safety experts have advocated the use of standardised processes. Efforts to mitigate the potential for errors and omissions during surgery have culminated in the development and use of strategies that have the potential to improve team performance, with a particular focus on communication. The introduction of safety checklists in surgery represents one strategy aimed at using a consistent approach in interdisciplinary team communications. However, despite the benefits associated with the use of checklists in surgery, universal implementation and compliance has been reported as being variable and inconsistent [4]. Further, there has been limited, if any, synthesis of the contextual issues associated with checklist introduction in surgery using an implementation science framework. In this review, we used a realist synthesis methodology to explain when, why, and how surgical safety checklist implementation adherence interventions work.

## Background

Over the last 10 years, the use of checklists in surgery has come into prominence. In 2008, the Surgical Safety Checklist (SSC) was a key outcome of the World Health Organization’s (WHO) *Safe Surgery Saves Lives* campaign. Since its introduction, the use of the WHO SSC has been mandated in operating rooms in over 4130 hospitals across 122 countries [5]. The original WHO SSC includes 19 items across three time-critical checkpoints: sign-in, timeout, and sign-out [6]. These checks are performed when the patient enters the OR, just prior to the procedure, and upon its completion. Checklist items require verbal confirmation by members of the surgical team of the completion of critical steps for ensuring the safe delivery of anaesthesia, antibiotic prophylaxis, availability of equipment, and other essential practices in surgery [6]. The intent of the checklist as a safety tool is to create a dialogue among team members, improving team communications and flattening the hierarchy that often characterises the culture of surgical teams [7, 8]. Checklists also function as an aide memoir for including key information or actions that may otherwise be overlooked or forgotten, thereby reducing the potential for human error [7, 9].

Improvements in team communications, a reduction in interruptions and distractions, and an increase in error identification and prevention of AE have been attributed to the use of surgical safety checklists [10, 11]. In terms of surrogate patient outcomes, there is persuasive evidence to support the effectiveness of checklists in relation to reducing mortality and complication rates following surgery. The results of several meta-analyses suggest that there is an association with checklist use and reductions in mortality [12, 13], wound infection [13, 14], pneumonia [14], blood loss [14], and any complication [13, 14].

Arguably, evidence of checklist effectiveness on outcomes is important [11, 15]; yet, there is often a disconnect between discussion of effectiveness and the manner and context in which the intervention was delivered. Evidence is also needed about the ways in which implementation of checklists vary in practice and across populations and healthcare settings [16]. This is especially crucial when the interventions are underpinned by behavioural change strategies and typically require multiple interactions over an extended period of time. These interactions have been described as being part of an ‘implementation chain’ that is only as strong as its weakest link [17]. Therefore, greater knowledge of the factors that affect each link of the chain may enable the chain to be strengthened. As such, uncovering these understandings may inevitably determine how a behaviour change intervention will work.

## Realist synthesis approach

The framework used to inform this synthesis on implementation and sustained use of checklists in surgery is Pawson’s [17] and Rycroft-Malone’s [18] realist evaluation approach. The realist approach is an emerging strategy for synthesising evidence and focuses on providing explanations for why implementation interventions may or may not work form whom, in what contexts, how and under what conditions, and *why* [18, 19]. Realist synthesis involves reviewing the evidence from complex interventions and assumes that no causal theory can always explain or predict outcomes in every context [20]. Thus, realism uses a contextually bound approach to causality: It is especially useful for exposing and disentangling the complexities of context and underlying interrelated mechanisms of implementation interventions [18]. Realism represents a major departure from conventional systematic reviews in that its goal is explanatory rather than summative [17]. This approach is well suited to undertaking a synthesis to develop explanatory models in areas where data are insufficient to identify and test relationships. The end product of the synthesis is not a summary of the evidence in support of relationships between the intervention and the outcome. Rather, the result is an explanatory model or candidate theory focusing on the characteristics of the intervention that makes it work (or not) in a given context and should allow implementation at the level of the mechanisms of action [18]. Complex behaviour-based interventions comprise theories, involve the actions of individuals, consist of an interrelated chain of steps or processes that interact and are rarely linear, are embedded in a social system, and are predisposed to modifications [21, 22]. As such, the underlying premise is that in a particular context, individuals are likely—but not always certain—to make similar choices. Consequently, specific contexts influence decisions that over time become emerging patterns of behaviour [17].

### Purpose

The purpose of our review was to explain when, why, and how implementation of surgical safety checklist interventions worked, or did not work well, *and why*. Specifically, our research question was as follows: *What aspects of checklist implementation determined success or failure in various situations and contexts and why?* We addressed this question through the identification and examination of underlying generative mechanisms (M) associated with the intervention, contexts, or conditions (C) under which the mechanisms operate and the outcomes (O) produced [4, 18]. That is, the action of the particular mechanism in a particular context will generate a particular outcome. Therefore, *if* the right processes operate in the right conditions *then* implementation will be sustained. Mechanisms have the potential to change minds and attitudes—and consequently, change behaviour [19]. To date, the theory underpinning healthcare professional behaviour change interventions is seldom explicated. In the context of surgery, it was not possible to unpack and explain the many intricacies associated with complex behaviour change interventions used to implement checklist use: Instead, we sought to gain insights and explanations that would be generalizable across multiple situations and contexts. As a starting point, we examined the implementation science literature on behaviour change interventions used in healthcare [16, 20, 21] and evaluated critically the evidence base that supports them, drawing on empirical data. The consequences of this synthesis were as follows:

1. Comparative analysis of approaches used to implement surgical safety checklists
2. Indicators to guide the choice of approaches that match the particular context
3. Indicators that may lead to the development of an explanatory model that can be used in implementation for healthcare organisations

## Methods

### Realist review process

The steps involved in the realist review process run parallel to conventional systematic reviews; however, in the former, these steps are iterative as opposed to being linear and sequential [22]. Rather than reviewing a focused literature addressing an a priori set of questions, the realist review itself generated some initial questions and led to the identification of more questions. Hence, a broad range of publications was needed to address questions about why, when, and how the intervention worked. The resultant model must be outcome focussed, uncovering ‘what works’ within different contexts and circumstances [22]. We adopted the framework proposed by Pawson [17, 19] and Rycroft-Malone et al. [22] which involves the following steps: scoping the review, searching and appraising the evidence, extracting and synthesising findings, and drawing conclusions and making recommendations.

### Search methods and data sources

A literature search dating back to 2008 was performed as checklist protocols such as the WHO SSC were developed and introduced from 2008 onwards. Articles were included if they were based on research and used either quantitative or qualitative methods, described the use of checklists in surgery, and focused on some aspect of checklist implementation (i.e. acceptability and/or compliance). Papers were excluded if they were not published as full-text articles, did not describe the process of implementation, or were based on commentaries or discussion papers. A combination of medical subject headings and key words included surgical checklist, safety checklist, WHO checklist, implementation, compliance, and adherence. Databases used in the search included PubMed, Cumulative Index of Nursing and Allied Health Literature (CINAHL), PsycINFO, and ProQuest Central. We stopped reviewing papers when data saturation had been reached.

### Middle-range theory identification

The middle-range theories we identified a priori provided a starting point in our efforts to explain what types of checklist implementation interventions work in surgery, for whom, and in what circumstances. The initial identification of middle-range theories in realist synthesis is essentially a speculative and iterative process [17, 23, 24]. As part of this process, we were also able to identify indicators that guided the choice of approaches used across contexts (consequence 2) and which would inform the development of an explanatory model (consequence 3). We initially identified theories which we believed to be evident to explicate the occurrence of certain outcomes. However, a necessary component of realist synthesis is to explore the applicability of these theories and, where appropriate, test their explanatory value [17]. During this iterative process, the validity of prospective middle-range theories was repeatedly questioned and refined, where appropriate. The included studies were again rechecked against the middle-range theories to establish which of the theories—if any—explained the outcome (i.e. sustained use of the safety checklist). In each paper, we sought data to test (i.e. refute, confirm, or refine) the middle-range theory by assessing its relevance and rigour [17]. Where the theories failed to explain the data, we sought new ones that would better explain data in the included studies. We applied the extracted evidence to these theories, iteratively refining our explanatory model to best explain the existing data. We generated broad themes and subthemes and listed propositions in relation to the delivery and sustainment of the implementation interventions.

We identified a priori, two middle-range theories to provide insights into the necessary processes for optimising the implementation and adoption of checklist protocols in practice. The first, Normalisation Process Theory (NPT), is useful in identifying factors that promote or inhibit the incorporation of complex intervention into clinical practice [25, 26]. The theory is also helpful in explaining how the intervention works, not only from early implementation but beyond the point where implementation becomes so much a part of clinical practice that it ‘disappears from view’, i.e. normalised. The four components of NPT include the following: coherence (sense-making), cognitive participation (engagement), collective action (actions that enable the intervention to be implemented), and reflexive monitoring (formal and informal assessment of the benefits and costs of the intervention) [25, 27]. As part of embedding a practice so that it becomes ‘normalised’ into routine work patterns, gaining an understanding of what health professionals do and how they work is crucial to inform how such practices can be sustained [26].

**Fig. 1:**
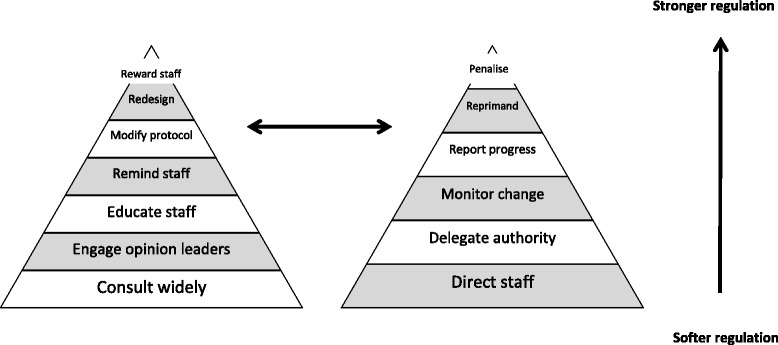
Pyramids of regulatory supports and sanctions, adapted from Braithwaite et al. [27]

In seeking further explanation for our emerging explanatory theory, we also drew on Braithwaite et al.’s [28] Responsive Regulation Theory (RRT). In RRT, ‘regulation’ refers to ‘steering the flow of events’ and encompasses strategies ranging from persuasion to enforcement that typically involve actors and multiple mechanisms. Responsive regulators use ‘softer’ mechanisms which traditionally rely on the premise that clinicians will change behaviour voluntarily and on self-regulation by professional groups [28]. These mechanisms are based on trust and respect as opposed to opting for immediate enforcement; nevertheless, there is necessarily the capacity to lead to sanction or even punishment. These mechanisms are mapped using twin complementary pyramids that symbolise either supports or sanctions. Figure 1 has been adapted from Braithwaite et al.’s [28] original model. This middle-range theory provides a nuanced approach given that implementation of complex behaviour change interventions in healthcare settings often requires both graduated and multiplex regulation [28].

### Quality appraisal

In realist synthesis, the reviewer ‘data mines’ each publication for evidence of its contribution to the development of the explanatory model [17]. Each piece of data is appraised for its utility and relevance, and the value of the evidence is based on its contribution to understanding of the questions in the review.

### Data abstraction and synthesis

As a precursor to data abstraction, we developed a data extraction tool to cover a broad range of information and to permit consolidation of the data across studies. Pawson [17] has warned against using such tools as they do not account for diversity in the data and, thus, may constrain flexibility. To address this potential, we ensured our extraction tool was broad enough to capture diversity in the research methods used. An overview of the information extracted on contextual factors that may affect the implementation process is presented as Additional file 1. We applied this template to the introduction, methods, results, and discussion sections of each report. Differences in coding were discussed with other team members and resolved by consensus.

### Explanatory focus

We focussed the review based on (i) the authors’ speculations about the barriers and facilitators to successful implementation; (ii) the authors’ speculations about the effects that characteristics of the intervention, context, and participants had on implementation; (iii) analysis of mediators or moderators that influenced implementation or outcomes (where mediators suggest *how* and *why* an intervention produces its effect and moderators suggest on *whom* or *under what conditions* an intervention produces its effect [29]); (iv) the ways in which the authors addressed challenges during the implementation process; and (v) participants’ reports of satisfaction, feasibility, and/or acceptability of implementation.

## Results

### Search outcomes

Thirty-five primary studies where checklist implementation and evaluation were described were included in the final analysis (Additional file 2). Data saturation was reached at 30 papers although we reviewed a further 5 papers to confirm that saturation was evident. Just under half (15/35) of these studies used either direct observations or audit or both, 11 others used surveys, and 7 were qualitative studies. The majority (31/35) of studies reported the implementation of the WHO Surgical Safety Checklist or an adaptation of this checklist protocol.

In keeping with realist synthesis methodology, we examined each step in the explanatory model separately, analysing data from all relevant publications that included data for that step [22]. Despite some limitations in data suitability in our included studies, the data available enabled us to broadly address the goals of the review. The types of interventions used in each study are detailed in Table 1. Where possible, we have drawn a comparative analysis of the approaches used to embed surgical safety checklists (consequence 1). Of the 35 studies included, 16 reported using educational sessions [18, 30–45]; however, there was limited, if any, detail about the number and duration of sessions (i.e. dose) offered or content covered in these sessions. There was also variability in the number of sessions staff were required to attend, and in some cases, particular professional disciplines (i.e. nurses) were required to attend, while other disciplines (i.e. physicians) were not. One study used an environmental redesign strategy in the form of interactive whiteboards for the ‘timeout’ section of the SSC checklist [46]. Over half (15/35) of the studies [18, 33, 35, 40, 42, 46–55] had ‘limited’ or ‘no’ implementation approach, i.e. there was either a perceived lack of awareness of a pre-planned implementation strategy or checklist introduction was not formally consolidated by any further implementation strategies. Although evidence of tailoring was described in only two studies [63, 64], the checklist was modified or adapted to context in 21 studies (where reported) [31–33, 36, 39, 40, 42–45, 49, 48, 51, 52, 54, 55, 57–59, 61, 63]. In the studies where compliance rates were reported, adherence for the check-in phase ranged from 23 to 98 % [18, 31, 32, 34, 36–39, 41, 45, 46, 48, 50, 52, 55, 56, 58] while timeout ranged from 19 to 100 % [18, 32, 36–39, 41, 42, 45, 46, 48, 50–52, 54, 58, 59], and sign-out ranged from 2 to 93 % [31, 32, 36, 37, 39, 41, 45, 46, 48, 50, 52, 55, 59]. Of the 35 studies, 8 reported attitudinal improvements in communication or safety culture [36, 38, 39, 45, 47, 49, 50, 53]. Follow-up periods to ascertain sustainability ranged from 1 to 12 months [31, 41, 42, 44, 50, 62].

**Table 1:** Implementation interventions and approaches used in each study

Implementation	Number of studies (*n* = 35)		Reported in
Types of interventions	*n* ^a^	%	
Opinion leaders	5	14.3	Bittle [62], Bohmer et al. [33], Conley et al. [60], Styer et al. [43], Yuan et al. [44]
Modelling	4	11.4	Bohmer et al. [33], Conley et al. [60], Haugen et al. [36], Norton and Rangel [45]
Widespread communication	10	28.6	Bashford et al. [31], Bell and Pontin [57], Berrisford et al. [58], Haugen et al. [36], Haynes et al. [37], Kearns et al. [50], Levy et al. [40], Norton and Rangel [45], Stryer et al. [43], van Klei et al. [61]
Educational sessions	16	45.7	Askarian et al. [30], Bashford et al. [31], Bliss et al. [32], Bohmer et al. [33], de Vries et al. [34], Gillespie et al. [35], Haugen et al. [36], Haynes et al. [37], Helmio et al. [38], Kwok et al. [39], Levy et al. [40], Anonymous [41], Sparkes and Rylah [42], Styer et al. [43], Kasatpibal et al. [18], Yuan et al. [44]
Self-assessment	1	2.8	Bashford et al. [31]
Clinical training	11	31.4	Bashford et al. [31], Berrisford et al. [58], Bittle [62], Calland et al. [47], Haugen et al. [36], Haynes et al. [37], Kwok et al. [39], Norton and Rangel [45], Sewell et al. [53], Vats et al. [54], Yuan et al. [44]
Audit and feedback	7	20.0	Bashford et al. [31], Berrisford et al. [58], Bittle [62], Bliss et al. [32], Haugen et al. [36], Truran et al. [51], van Klei et al. [37]
Environmental redesign	1	2.8	Mainthia et al. [46]
Rewards/incentives	0	0	-
Coercion	0	0	-
Restrictions/sanctions	0	0	-
Performance data	8	22.8	Askarian et al. [30], Berrisford et al. [58], Bliss et al. [32], de Vries et al. [34], Haynes et al. [37], Kwok et al. [39], Sewell et al. [53], Yuan et al. [44]
Approach			
Planned	16	45.7	Askarian et al. [30], Bashford et al. [31], Bell and Pontin [57], Berrisford et al. [58], Bittle [62], Bliss et al. [32], Conley et al. [60], Haugen et al. [36], Haynes et al. [37], Helmio et al. [38], Kwok et al. [39], Norton and Rangel [45], Anonymous [41], Stryer et al. [43], van Klei et al. [61], Yuan et al. [44]
Limited/none	15	42.8	Bohmer et al. [33], Calland et al. [47], Gillespie et al. [35], Levy et al. [40], Mainthia et al. [46], Pérez-Guisado [48], Sparks and Rylah [42], Takala et al. [49], Kearns et al. [50], Sewell et al. [53], Rydenfalt et al. [52], Truran et al. [51], Vats et al. [54], Vogts et al. [55], Kasatpibal et al. [18]
Evidence of tailoring	2	5.7	Foucade et al. [63], Russ et al. [64]

^a^Not all studies reported implementation strategies used

### Application of the data to the explanatory model

We applied the evidence synthesised through this review to the Normalisation Process Theory (NPT) framework with the results presented in Additional file 3. Our findings suggest that implementation of safety checklists in surgery had limited coherence for participants, particularly for physicians, many of whom believed that the formal introduction of a checklist was redundant as they were already enacting these principles in practice. Participants were unable to perceive the overall benefits to team members and patients, despite the intent of the checklist being focussed on the deliberate communication of information to all team members. In the majority of review studies, there was limited engagement (i.e. cognitive participation) as evidenced by variations in checklist item usage rates, and this was often discipline-specific. This culminated in low collective action because of the perceived inconvenience associated with checklist implementation and the added workload this imposed. There was, however, robust evidence of reflexive monitoring as participants reported work process improvements (e.g., venous thromboembolism/antibiotic prophylaxis rates) and enhanced safety culture, morale, and satisfaction. Yet in those studies that reported longitudinal data on checklist compliance, there was evidence of intervention decay in consistency in checklist completion rates.

In testing the relevance of the Responsive Regulation Theory (RRT) to the data extracted, we found robust evidence for the use of implementation interventions that reflected ‘softer’ regulatory strategies of support (and/or reward) rather than ‘sanction’ (and/or punishment). In 16/35 studies, participants were supported with the requisite education in the use of a checklist protocol. Study site hospitals ran in-service programmes (e.g. lectures/presentations), and some conducted multiple training sessions alongside dissemination through local newsletters and posters. Five studies reported enlisting opinion leaders to promote checklist use among staff. Insofar as using regulatory strategies from the sanction pyramid (Fig. 1), eight review studies reported monitoring relative to clinical audit (i.e. compliance) and published performance data (i.e. clinical or adverse incident rates). Notably, 100 % compliance with specific subsections (e.g. timeout) or items was reported in only two studies [40, 59]. In Levy et al.’s [40] study, compliance rates were drawn retrospectively from the electronic medical record.

### Explanatory model

During the process of theory synthesis, we generated propositions to further refine our explanatory model. These four propositions focussed on the identification of factors that impact on the mechanism (process) of checklist implementation relative to tailoring to context, practice fidelity, and sustainment. Table 2 presents the propositions, the mechanism of implementation, and the alignment of these mechanisms with the emergent explanatory theory and the identified middle-range theories.

**Table 2:** Propositions used to further refine the explanatory model

Proposition	Mechanism of implementation	Coherence with middle-range theory (supporting data from review studies)
Checklist protocols that are prospectively tailored to the context are more likely to be used and sustained in practice.	Process simplification	Normalisation Process Theory
	• Keeping it simple	
	• Modifying to reflect workflow	
	• Tailoring to context	
	Reflection	
	• Collective learning	
	• Monitoring	
	• Feeding back	
Fidelity and sustainability is increased when checklist protocols can be seamlessly integrated into daily professional practice.	Process simplification	Normalisation Process Theory
	• Keeping it simple	
		Responsive Regulation Theory
	• Modifying to reflect workflow	
	• Tailoring to context	
	Reflection	
	• Collective learning	
	• Monitoring	
	• Feeding back	
Routine embedding of checklist protocols in practice is influenced by factors that promote or inhibit clinicians’ participation.	Active leadership	Responsive Regulation Theory
	• Discipline leader	
		Normalisation Process Theory
	• Frontline decision-making	
	• Active participation	
	Support strategies	
	• Controlled roll-out	
	• Support without sanction	
	• Communicating the message	
	Process simplification	
	• Keeping it simple	
	• Modifying to reflect workflow	
	• Tailoring to context	
	Reflection	
	• Collective learning	
	• Monitoring	
	• Feeding back	
Regulation reinforcement mechanisms that are more contextually responsive should lead to greater compliance with using checklist protocols.	Active leadership	Responsive Regulation Theory
	• Discipline leader	
	• Frontline decision-making	
	• Active participation	
	Support strategies	
	• Controlled roll-out	
	• Support without sanction	
	• Communicating the message	

#### Proposition 1: Checklist protocols that are prospectively tailored to the context are more likely to be used and sustained in practice

There is inadequate data in relation to the implementation process (i.e. rationale for selection of interventions), tailoring (i.e. barrier analysis and protocol modifications), and sustainment (i.e. longitudinal follow-up compliance data). In the review studies, most checklists were reportedly modified to reflect current work practices, but the specific nature of these modifications was not detailed. In three review studies [45–47], participants specifically adapted the checklist protocol to their particular context (i.e. paediatric, general, or laparoscopic surgeries) and had either revised specific items to make them more relevant or had used novel interactive implementation strategies, e.g. electronic whiteboard with traffic light colours to denote that the checks were carried out. These strategies culminated in substantial increases in core item compliance rates post-implementation ranging from 50 to 82 % [46]. Real-world compliance with checklists varies: In one hospital in the Netherlands, completion rates were as low as 39 % [61]; while a recent Canadian study [65] found that self-reported compliance rates across 101 hospitals were high, ranging from 92 to 98 % [63]. In many of the review studies, early checklist implementation culminated in team-based checks as opposed to checks that were performed by individuals alone. In 16/35 studies, participants reported improvements in work processes (i.e. risk identification, contingency planning), safety climate, teamwork, and communication [18, 33, 36–39, 45, 47, 49, 50, 53, 54, 57–59, 62]. In contrast with these findings, Truran et al. [51] reported a reduction in venous thromboembolism compliance following checklist implementation. In those studies where longitudinal data were collected over 6–12 months, usage patterns were variable and compliance rates across some checklist items waned. As none of the review studies reported how implementation may have been influenced by the presence or absence of broader contextual events (e.g. organisational restructuring and changes in clinical leadership), their impacts on implementation and sustainability are unknown.

#### Proposition 2: Fidelity and sustainability is increased when checklist protocols can be seamlessly integrated into daily professional practice

We found limited support for this proposition as implementation was not sufficiently reported in the review studies to speculate about fidelity. Despite an intervention being deemed to be demonstrably efficacious, its long-term impact is contingent on its effectiveness in the ‘real world’, that is, the clinical environment [67–69]. Obstacles such as workload and the complexity of role interdependency shape the context in which surgical teams work [70]. In the complex arsenal of safety improvement initiatives, Bosk [71] warns against the assumption that the ‘simple checklist’ as a technical/adaptive solution will necessarily address broader contextual issues—i.e. the sociocultural problems. Sustainability in safety improvement clearly needs organisational and professional commitment to a wider set of interventions concerning safety in the surgical process [65, 71, 72]. Our findings suggest that the success of embedding a new or innovative clinical practice in surgery is especially challenging when it is being implemented against the backdrop of team culture: that is, organisational and operating room norms and expectations, and the surgical team, a tripartite composed of health professionals from disparate disciplinary orientations and practice paradigms [8]. Clearly, the ways in which safety improvement initiatives such as checklists are embedded in clinical practice has consequences—and their implementation can culminate in their misuse or non-use [4]. In most of our review studies, checklists were incompletely or inconsistently executed.

#### Proposition 3: Routine embedding of checklist protocols in practice is influenced by factors that promote or inhibit clinicians’ participation

There was moderate support for this proposition. Factors such as forgetfulness, time constraints, and duplication were identified as barriers to implementation success, [34, 53, 63, 64] but they may actually be surrogates for a perceived lack of value for the practice. In several of our review studies, checklist implementation posed logistical challenges in relation to workflow, especially during emergency procedures [35, 50, 55, 63, 64]. In one study [52], nursing staff were not observed to participate in the ‘timeout’ section of the checklist as they were already engaged in performing other intraoperative tasks such as draping the patient. The study authors speculated that the lack of nurses’ compliance in this process was related to gaps in their understanding of the intent of the checklist as a risk management tool. Aspects of context such as organisational culture, professional identity, workload, and professional identity were recognised in several review studies [35, 42, 43, 63] and in relation to being either barriers or enablers to checklist implementation [63, 64]. Undoubtedly, a mandatory checklist can be difficult or even impossible to implement without the support from senior leaders within the organisation [71].

#### Proposition 4: Regulation reinforcement mechanisms that are more contextually responsive should lead to greater compliance in using checklist protocols

There was partial support for this proposition. Most implementation interventions occurred in relation to supports provided and focussed on engaging health professionals rather than sanctioning them. There were no serious sanctions evident in any of the studies—perhaps because surgical teams function interdependently and so rely on the interdisciplinary knowledge of others within the team. Conceivably, any form of serious sanction may impact on team dynamics and professional relations. On the sanction pyramid, ‘soft’ regulation in the form of monitoring compliance through audit and reporting was evident in several studies [54, 56, 58, 62]. Although this mechanism of sanction proved to be reasonably effective, the reporting processes (i.e. publication of non-compliant individuals’ names) used in these studies was not explicit. As such, it is impossible to speculate about whether checklist implementation vis-à-vis sustained compliance and patient/team outcomes would have been better than those reported. Notably, non-compliance in checklist use was not interpreted by participants as a deliberate violation of any agreed standard. No cases of serious disciplinary sanctions, e.g. reprimand, suspension, or dismissal for refusal to follow a protocol directive, were known or reported in any of the review studies. In Australia, as in countries such as the United States of America (USA), financial penalties apply to some adverse events (e.g. pressure injuries). However, unlike the USA where the ‘Universal’ checklist protocol is mandated for hospitals seeking accreditation by the US Joint Commission, the Australian hospital accreditation agency currently has a non-mandatory standard.

The body of literature on checklists reflects the growing debate about managing the issues around the unintended consequences of their mandatory introduction in surgery. For instance, Urbach and colleagues [65] conducted a population-based survey of 101 Ontarian hospitals and found that outcomes changed significantly—for better or worse. The authors of that study concluded that checklist use did not culminate in striking improvements in patient outcomes, a finding that runs counter to other studies [37, 61, 70] in this field. In an earlier qualitative study, Thommasen et al. [72] found that using checklists was perceived as having the opposite effect for which they were intended. That is, rather than focussing on the patient, using the checklist actually diverted team members’ attention away from the patient. While checklists are specifically intended to improve communication between team members and their patients, they can also act as distractions or may even lead to complacency. Complacency is the biggest threat to patient safety particularly when it is the prevailing belief of healthcare organisation leaders that mandating checklists offers the panacea [71]. A chilling reminder of the unintended effects of checklist use is wrong site surgery, which persists despite mandating their use.

### Interaction of contexts and causal mechanisms on implementation and sustainment

**Fig. 2:**
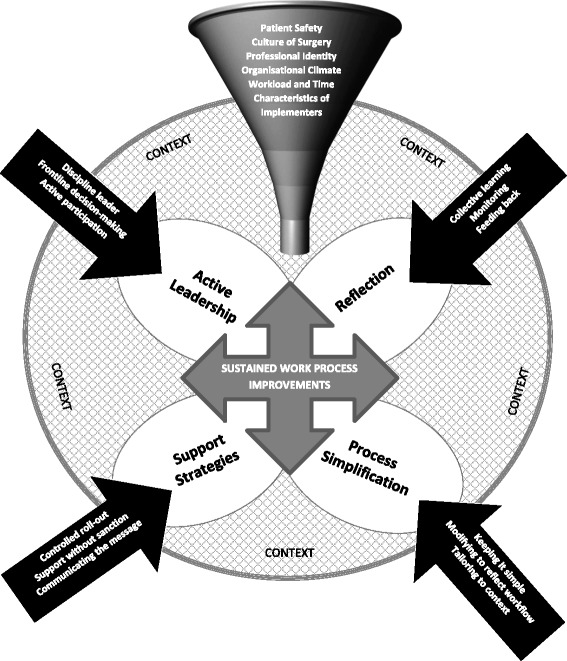
Explanatory model for implementation and sustainment of checklists in surgery

Pawson and Tilley’s [24] *context + mechanism → outcome* framework was applied to describe the complex relationships between the causal mechanisms and the effects that particular contexts have on their implementation and the outcome. Additional file 4 illustrates the CMO configuration of checklist implementation and sustainability in surgery. The emergent explanatory model is illustrated in Fig. 2. Factors that feed into the *context* include the need for patient safety, culture of surgery, professional identity, organisational and departmental factors, workload and time, and characteristics of the implementers (i.e. practice paradigm, knowledge/skills, beliefs, memory). Success or failure in implementation of any new or innovative practice in surgery is determined by the synergy of these omnipresent contextual factors, which can act either as barriers or enablers to implementation and sustainment of checklist protocols. In realist terms, there are multiple mechanisms that influence the outcome [19]; the *mechanisms* through which checklist implementation is operationalised include four processes: *active leadership*, *support strategies*, *process simplification*, and *reflection*. The interaction of these mechanisms and the interventions that support them (Fig. 2, text in the black arrows) operating to various degrees, depending on context to yield the *outcome*, sustained work process improvement.

*Active leadership* mechanisms that encourage leadership, decision-making at the point of care, and team participation are moderated by organisational climate, professional identity, and the characteristics of the implementers. In contexts where checklist protocols were introduced and led by physicians, work process improvements were more likely to be sustained in practice [39, 58]. When implementation was devolved to other team members such as nurses, protocol fidelity and sustainability were limited [35, 40]. Mechanisms that encompass *support strategies* are designed *to* engage stakeholders in dialogue and support rather than threaten and encourage learning. Yet, the poise between using ‘softer’ versus ‘harder’ regulation processes is tenuously balanced and is contingent on contextual factors such as organisational and departmental expectations, norms, and safety climate. Ultimately, regulation is controlled by broader societal norms and expectations [28, 71], and therefore, checklist implementation will necessarily require modification from time to time. *Process simplification* involves tailoring the checklist protocol to the nuances of the context, which ensures that practice sustainment over time will be more likely. However, success or failure is contingent on contextual factors such as organisational climate, culture of surgery, and workload and time [73].

## Discussion

### Summary of main findings

Through the use of realist evaluation methodology, we have uncovered how various types of surgical safety checklist protocols are being used in the reality of the clinical setting. In doing so, the findings from this synthesis have increased understanding of the ways in which implementation interventions may bring about behaviour change. Ours is the first realist synthesis to examine the implementation and sustained use of checklist protocols in surgery. Two consequential and meaningful findings have emerged: First, the sustained use of surgical checklists is discipline-specific and is more successful when medical staff are actively participating and leading the implementation process. Second, involving clinicians in tailoring the checklist to the nuances of their context and encouraging them to reflect on and evaluate the implementation process enable greater participation and ownership. The degree of success or failure in implementation and sustainment relies on sociocultural factors, the ways in which individuals respond, their reasoning, and the resources they have available—all of which obviously vary [74]. Implementation of behavioural interventions designed for practice improvement is rarely, if ever, completely successful or unsuccessful, having patches of success or failure. Across our review studies, implementation of checklist protocols led to improved work process practices and teamwork in some settings but failed or had limited success in others. Conceivably, the chain of implementation is only as strong as its weakest link [17]: In implementing surgical checklists, the weakest link in the chain appears to include leadership, tailoring, and reflection on the process. Undoubtedly, context and mechanism factors that underpin the implementation chain are inexorably intertwined and either support or inhibit the attainment of desired outcomes [17].

### The crucial role of context

In the implementation of safety improvement initiatives, the criticality of *context* in successful implementation cannot be ignored. It is essential to determine the effectiveness of these initiatives relative to particular clinical settings [74, 75]. In addition to questions of effectiveness (i.e. whether, how, and why), implementers must consider the unintended adverse consequences of implementing safety practices [71, 74]. In order to evaluate the effectiveness of safety improvement initiatives across contexts, it is imperative to describe systematically each context to determine the ways in which it is similar and different from others. As part of the evaluation process, implementers must also ascertain to what extent these similarities and differences might impact on the effectiveness of the practice being implemented [74]. The explanatory theory developed through this realist synthesis extends beyond the focus of implementation and its causal chain to include an understanding of contextual factors and how they help or hinder implementation of surgical, safety checklists as just one of many safety improvement initiatives. Theory has not commonly been used in the field of safety research to inform implementation [75, 76]. None of the studies included in our synthesis proposed explicit theory or logic to explain why implementation of a checklist protocol should work. Theory applied in safety improvement research can help to explain clinical and organisational behaviour (e.g. compliance/non-compliance), tailoring of interventions to a given problem or context, and evaluating implementation and the mechanism of action [74, 75].

### Selection of interventions

Many of the interventions used to change professional behaviour in our review had modest and variable effects which may be attributed to the ways in which the interventions were selected. Selection of interventions across these studies appeared often to be based on disciplinary discretion rather than on an explicit rationale that takes targeted behaviour and context into account based on identified barriers and enablers. Michie and colleagues’ [16, 79] Theoretical Domains Framework (TDF) provides researchers, clinicians, and policy makers with a coherent guide to selecting behaviour change interventions based on identifying contextual barriers and enablers. The TDF is a systematic method of selection which improves intervention design because it incorporates a wide-ranging understanding of the nature of the behaviour that requires change, the context, and the practitioners involved [16]. Standardised behaviour change interventions that use a ‘top down’ approach may lack flexibility to respond to local barriers and circumstances [77]. Moreover, in implementation, it is not about *what* is done but more about *how* all of the strategies interact in the multifaceted intervention [20, 78]. For the studies included herein, it is unclear *what* component (i.e. active ingredient) of the intervention affected the change in individuals’ practice behaviours vis-à-vis checklist use. The intensity of the intervention ‘dose’ was also unclear.

### Tailoring

Tailoring will accommodate context but should also address barriers. In the studies we reviewed, tailoring occurred at one level: checklist modification to make it ‘local’ in response to context. Checklist tailoring may involve rationalising the items on the checklist to be more specific to the surgical specialty, e.g. hospital that only does ophthalmology, and thus, where appropriate, such modifications are more likely to lead to sustained use. In Australia and New Zealand, several peak professional organisations (e.g. Royal Australasian College of Surgeons, Australian College of Operating Room Nurses, Australian and New Zealand College of Anaesthetists) have developed modified versions of the WHO SSC. These efforts acknowledge the importance of tailoring the checklist to the local conditions of the organisation. However, changes over time can render a well-designed intervention irrelevant if professional practice standards change or legislation is introduced [66, 79]. Hence, prior to implementation, it is important for interventionists to consider likely changes and implications for those whom the intervention is designed [79, 80]. Despite the growing imperative to cultivate health consumer engagement [16, 79], health consumers, being the recipients of care, do not appear to be engaged in the introduction and tailoring of checklist protocols in surgery, albeit that patients undergoing surgery may potentially influence implementation. Clearly, there is an increasing need to understand the role that health consumers have in identifying/developing interventions targeted to effect behaviour change.

### Implementation fidelity and sustainability

A safety checklist should be implemented to align with its intentions, i.e. to improve work processes through better team communications. Being realistic in implementation means focussing on what can be done and setting aside what may seem desirable but not feasible [80]. Ideally, fidelity of implementation should centre on factors that enable work process improvements. In some review studies, process adherence was evaluated longitudinally but had, over time, decayed. Our findings support the notion that flexibility in process and reflexivity seem integral to sustainability. In an earlier Cochrane review, 26 randomised controlled trials found that interventions that had been tailored to address contextual barriers to change were more likely to improve professional practice compared with no intervention or dissemination of guidelines [20]. However, most methods to develop tailored interventions need further development. Our synthesis has revealed that in most instances, the interventions were implemented inconsistently, rendering their impact and overall sustainment limited. It is important to garner staff perceptions of barriers to enable tailoring of interventions to the context [78, 79].

### Strengths and limitations

We recognise that this synthesis has some limitations. First, the quality of the review is only as robust as the primary studies on which the synthesis is based. A major constraint is that the majority of the studies included in our review gave only a cursory description of the implementation strategies used to embed surgical safety checklists in practice. The lack of data in this regard imposes caveats on the extent to which all aspects of the explanatory theories identified herein can be applied to the clinical contexts where checklist implementations occurred. Most of the papers included did not provide information on the fidelity of intervention delivery. Perhaps richer and more detailed descriptions of the implementation process used would have permitted a more fine-grained analysis of the contextual barriers and enablers to implementation. Subsequently, testing our middle-range theories and propositions may have resulted in findings with fewer nuances. Moreover, in answering the same research questions, a different group of researchers may have inevitably identified a different set of primary sources and made different judgements about their quality and relevance. Arguably, this is an inherent characteristic in any realist review that addresses the nuances of implementing complex interventions across different organisational, social, or environmental contexts. Importantly, the reviewer’s interpretive judgements are integral to the synthesis process and can never fully be standardised or rationalised [23, 66].

## Conclusions and recommendations

Rather than attempting to control contextual factors, they need to be harnessed because they inevitably determine the mechanisms that are activated to generate the outcomes. Despite that the explanatory model generated herein is in its early stages of development, it may be used to inform areas where further translational research in this area is needed. Through the use of realist evaluation methodology, we envisage these findings may assist clinical leaders and hospital administrators to select appropriate interventions to their particular context. We recommend that future researchers test the explanatory model developed through this synthesis specifically focussing on yielding more data on checklist implementation, viz undertaking process evaluations and designing studies across multiple healthcare settings and then collecting data on how implementation varied across contexts. The explanatory model developed herein is intended to provide the evidence needed to strengthen each link in the implementation chain.

## Additional files

Additional file 1:
**Data synthesis: categories of information extracted from primary reports.**
Additional file 2:
**Feasibility/acceptability/fidelity studies of surgical checklist implementation interventions (** ***n*** **= 35).**
Additional file 3:
**NPT analysis for evaluation of checklist implementation in surgery.**
Additional file 4:
**CMO configuration in relation to sustainability of checklists in surgery.**

## References

[1] de Vries E, Ramrattan M, Smorenburg S, Gouma D, Boermeester M (2008). The incidence and nature of in-hospital adverse events: a systematic review. Qual Saf Health Care.

[2] Yii M, Ng K (2002). Risk-adjusted surgical audit with the POSSUM scoring system in a developing country. Physiological and operative severity score for the enumeration of mortality and morbidity. Br J Surg.

[3] McConkey S (2002). Case series of acute abdominal surgery in rural Sierra Leone. World J Surg.

[4] Weiser T, Berry W (2013). Review article: perioperative checklist methodologies. Can J Anesth.

[5] Surgical safety web map. [http://gis.harvard.edu/services/products/surgical-safety-web-map]

[6] WHO (2008). Implementation of the surgical safety checklist.

[7] Low D, Walker I, Heitmiller E (2012). Implementing checklists in the operating room. Paediatr Anaesth.

[8] Gillespie B, Chaboyer W, Longbottom P, Wallis M (2010). The impact of organisational and individual factors on team communication in surgery: a qualitative study. Int J Nurs Stud.

[9] Winters BD, Gurses AP, Lehmann H, Sexton JB, Rampersad CJ, Pronovost PJ (2009). Clinical review: checklists - translating evidence into practice. Crit Care.

[10] Birkmeyer J (2010). Strategies for improving surgical quality--checklists and beyond. N Engl J Med.

[11] Spiess B (2013). The use of checklists as a method to reduce human error in cardiac operating rooms. Int Anesthesiol Clin.

[12] Borchard A, Schwappach D, Barbir A, Bezzola P (2012). A systematic review of the effectiveness, compliance, and critical factors for implementation of safety checklists in surgery. Ann Surg.

[13] Bergs J, Hellings J, Cleemput I, Zurel O, De Troyer V, Van Hiel M (2014). Systematic review and meta-analysis of the effect of the World Health Organization surgical safety checklist on postoperative complications. Br J Surg.

[14] Gillespie B, Chaboyer W, Thalib L, Fairweather N, Slater K (2014). Effect of using a safety checklist in surgery on patient complications: a systematic review and meta-analysis. Anaesthesiology.

[15] Walker A, Reshamwalla S, Wilson I (2012). Surgical safety checklists: do they improve outcomes?. BJA.

[16] Michie S, van Stralen M, West R (2011). The behaviour change wheel: a new method for characterising and designing behaviour change interventions. Implement Sci.

[17] Pawson R (2006). Evidence-based policy: a realist perspective.

[18] Kasatpibal N, Senaratana W, Chitreecheur J, Chitirosniramit N, Pakvipas P, Junthascopeepan P (2012). Implementation of the World Health Organization surgical safety checklist at a university hospital in Thailand. Surg Infect (Larchmt).

[19] Pawson R, Manzano-Santaella A (2012). A realist diagnostic workshop. Evaluation.

[20] Baker R, Camosso-Stefinovic J, Gillies C, Shaw E, Cheater F, Flottorp S, et al. Tailored interventions to overcome identified barriers to change: effects on professional practice and health care outcomes. Cochrane Database of Systematic Reviews 2010, Issue 3. Art. No.: CD005470. doi:10.1002/14651858.CD005470.pub2. 10.1002/14651858.CD005470.pub2 PMC4164371 20238340

[21] Campbell N, Murray E, Darbyshire J, Emery J, Farmer A, Griffiths F (2007). Designing and evaluating complex interventions to improve health care. BMJ.

[22] Rycroft-Malone J, McCormack B, Hutchinson A, DeCorby K, Bucknall T, Kent B (2012). Realist synthesis: illustrating the method for implementation research. Implement Sci.

[23] Pawson R, Greenhalgh T, Harvey G, Walshe K (2005). Realist review--a new method of systematic review designed for complex policy interventions. J Health Serv Res Policy.

[24] Pawson R, Tilley N (1997). Realistic evaluation.

[25] May C, Finch T (2009). Implementation, embedding, and integration: an outline of Normalization Process Theory. Sociology.

[26] May C (2006). Mobilising modern facts: health technology assessment and the politics of evidence. Sociol Health Illn.

[27] May C, Mair F, Finch T, MacFarlane A, Dowrick C, Treweek S (2009). Development of a theory of implementation and integration: Normalization Process Theory. Implement Sci.

[28] Braithwaite J (2011). The essence of responsive regulation. UBC Law Rev.

[29] Kraemer H, Lowe K, Kupfer D (2005). To your health: how to understand what research tells us about risk.

[30] Askarian M, Kouchak F, Palenik CJ (2011). Effect of surgical safety checklists on postoperative morbidity and mortality rates, Shiraz, Faghihy Hospital, a 1-year study. Qual Manag Health Care.

[31] Bashford T, Reshamwalla S, McAuley J, McNatt Z, Gebreedhen YD. Implementation of the WHO Surgical Safety Checklist in an Ethiopian Referral Hospital. Patient Safety in Surgery. 2014;8:16. 10.1186/1754-9493-8-16 PMC4022152 24678854

[32] Bliss LA, Ross-Richardson CB, Sanzari LJ, Shapiro DS, Lukianoff AE, Bernstein BA (2012). Thirty-day outcomes support implementation of a surgical safety checklist. J Am Coll Surg.

[33] Bohmer A, Wappler F, Tinschmann T, Rixen D, Bellendir M, Schwanke U (2012). The implementation of a perioperative checklist increases patients’ perioperative safety and staff satisfaction. Acta Anaesthesiol Scand.

[34] de Vries EN, Hollmann MW, Smorenburg S, Gouma DJ, Boermeester MA (2009). Development and validation of the SURgical PAtient Safety System (SURPASS) checklist. Qual Saf Health Care.

[35] Gillespie B, Chaboyer W, Wallis M, Fenwick C (2010). Why isn’t time out being implemented? An exploratory study. Qual Saf Health Care.

[36] Haugen A, Søfteland E, Eide G, Sevdalis N, Vincent C, Nortvedt M (2013). Impact of the World Health Organization’s surgical safety checklist on safety culture in the operating theatre: a controlled intervention study. Br J Anaesth.

[37] Haynes AB, Weiser TG, Berry W, Lipsitz S, Breizat A, Dellinger E (2009). A surgical safety checklist to reduce morbidity and mortality in a global population. N Engl J Med.

[38] Helmio K, Takala R, Aaltonen A, Katila A, Peltomaa K, Ikonen T (2011). First year with WHO Surgical Safety Checklist in 7148 otorhinolaryngological operations: use and user attitudes. Clin Otolaryngol.

[39] Kwok A, Funk L, Baltaga R, Lipsitz S, Merry A, Dziekan G (2012). Implementation of the World Health Organization surgical safety checklist, including introduction of pulse oximetry, in a resource-limited setting. Ann Surg.

[40] Levy S, Senter C, Hawkins R, Zhao J, Doody K, Kao L (2012). Implementing a surgical checklist: more than checking a box. Surgery.

[41] Anonymous. Communicating for success: Royal Bolton Hospital NHS Foundation Trust introduces the Surgical Safety Checklist. J Perioper Pract 2010;20:85–86. 20642232

[42] Sparkes D, Rylah B (2010). The World Health Organization surgical safety checklist. Br J Hosp Med (Lond).

[43] Styer K, Ashley S, Schmidt I, Zive E, Eappin S (2011). Implementing the World Health Organization surgical safety checklist: a model for future perioperative initiatives. AORN J.

[44] Yuan CT, Walsh D, Tomarken JL, Rachelle A, Shakpeh J, Bradley EH (2012). Incorporating the World Health Organization surgical safety checklist into practice at two hospitals in Liberia. Jt Comm J Qual Patient Saf.

[45] Norton E, Rangel S (2010). Implementing a pediatric surgical safety checklist in the OR and beyond. AORN J.

[46] Mainthia R, Lockney T, Zotov A, France DJ, Bennett M, St Jacques PJ (2012). Novel use of electronic whiteboard in the operating room increases surgical team compliance with pre-incision safety practices. Surgery.

[47] Calland J, Turrentine F, Guerlain S (2011). The surgical safety checklist: lessons learned during implementation. Am Surg.

[48] Pérez-Guisado J (2012). Implementation of the World Health Organization surgical safety checklist in plastic and reconstructive patients. Plast Reconstr Surg.

[49] Takala R, Pauniaho S, Kotkansalo A, Helmio K, Blomgren M, Helminan M (2011). A pilot study of the implementation of WHO surgical checklist in Finland: improvements in activities and communication. Acta Anaesthesiol Scand.

[50] Kearns J, Uppal V, Bonner J, Robertson J, Daniel M, McGrady E (2011). The introduction of a surgical safety checklist in a tertiary referral obstetric centre. BMJ Qual Saf.

[51] Truran P, Critchley R, Gilliam A (2011). Does using the WHO surgical checklist improve compliance to venous thromboembolism prophylaxis guidelines?. Surgeon.

[52] Rydenfält C, Johansson G, Odenrick P, Åkerman K, Larsson PA (2013). Compliance with the WHO surgical safety checklist: deviations and possible improvements. Int J Qual Health Care.

[53] Sewell M, Adebibe M, Jayakumar P, Jowett C, Kong K, Vemulapalli K (2011). Use of the WHO surgical safety checklist in trauma and orthopaedic patients. Int Orthop.

[54] Vats A, Vincent C, Nagpal K, Davies R, Darzi A, Moorthy K (2010). Practical challenges of introducing WHO surgical checklist: UK pilot experience. BMJ.

[55] Vogts N, Hannam J, Merry A, Mitchell S (2011). Compliance and quality in administration of a surgical safety checklist in a tertiary New Zealand hospital. N Z Med J.

[56] de Vries E, Prins H, Crolla R, den Outer A, van Andel G, van Helden S, et al. Effect of a comprehensive surgical safety system on patient outcomes. N Engl J Med 2010, 363:1928–37. 10.1056/NEJMsa0911535 21067384

[57] Bell R, Pontin L (2010). How implementing the surgical safety checklist improved staff teamwork in theatre. Nurs Times.

[58] Berrisford R, Wilson I, Davidge M, Sanders D (2012). Surgical time out checklist with debriefing and multidisciplinary feedback improves venous thromboembolism prophylaxis in thoracic surgery: a prospective audit. J Cardiothorac Surg.

[59] Cullati S, Licker MJ, Francis P, Degiorgi A, Bezzola P, Courvoisier DS (2014). Implementation of the surgical safety checklist in Switzerland and perceptions of its benefits: cross-sectional survey. PLoS One.

[60] Conley D, Singer S, Edmondson L, Berry WR, Gawande AA. Effective surgical safety checklist implementation. J Am Coll Surg. 2011;212:873-79. 10.1016/j.jamcollsurg.2011.01.052 21398154

[61] van Klei WA, Hoff RG, van Aarnhem EEHL, Simmermacher RKJ, Regli LPE, Kappen TH (2012). Effects of the introduction of the WHO “surgical safety checklist” on in-hospital mortality: a cohort study. Ann Surg.

[62] Bittle M (2011). Theatre team learns to use checklist to make surgery safer. Nurs N Z.

[63] Fourcade A, Blache JL, Grenier C, Bourgain JL, Minvielle E. Barriers to staff adoption of a surgical safety checklist. BMJ Qual Saf. 2012;21(3):191–197. 10.1136/bmjqs-2011-000094 PMC3285141 22069112

[64] Russ SJ, Sevdalis N, Moorthy K, Mayer EK, Rout S, Caris J, et al. A Qualitative Evaluation of the Barriers and Facilitators Toward Implementation of the WHO Surgical Safety Checklist Across Hospitals in England Lessons From the “Surgical Checklist Implementation Project”.Ann Surg. 2015;261(1):81–91. doi:10.1097/SLA.0000000000000793 10.1097/SLA.0000000000000793 25072435

[65] Urbach D, Govindarajan A, Saskin R, Wilton A, Baxter N (2014). Introduction of surgical safety checklist in Ontario, Canada. N Engl J Med.

[66] Greenhalgh T, Robert G, Macfarlane F, Bate P, Kyriakidou O, Peacock R (2005). Storylines of research in diffusion of innovation: a meta-narrative approach to systematic review. Soc Sci Med.

[67] Murray E, Treweek S, Pope C, MacFarlane A, Ballini L, Dowrick C (2010). Normalisation process theory: a framework for developing, evaluating and implementing complex interventions. BMC Med.

[68] Greenhalgh T, Peacock R (2005). Effectiveness and efficiency of search methods in systematic reviews of complex evidence: audit of primary sources. BMJ.

[69] Greenhalgh T, Robert G, Macfarlane F, Bate P, Kyriakidou O (2004). Diffusion of innovations in service organizations: systematic review and recommendations. Milbank Q.

[70] Gillespie BM, Gwinner K, Fairweather N, Chaboyer W (2013). Building shared situational awareness in surgery through distributed dialog. J Multidiscip Healthc.

[71] Bosk C, Dixon-Woods M, Pronovost PJ (2009). The art of medicine reality check for checklists. N Engl J Med.

[72] Thomassen Ø, BrattebøJon G, Heltne K, Søfteland E, Espeland A (2010). Checklists in the operating room: help or hurdle? A qualitative study on health workers’ experiences. BMC Health Serv Res.

[73] Gillespie B, Gwinner K, Chaboyer W, Fairweather N (2013). Team communications in surgery—creating a culture of safety. J Interprof Care.

[74] Shekelle P, Pronovost P, Wachter R, Taylor S, Dy S, Foy R (2010). Assessing the evidence for context-sensitive effectiveness and safety of patient safety practices: developing criteria.

[75] Gagliardi A, Alhabib S (2015). Trends in guideline implementation: a scoping systematic review. Implement Sci.

[76] Michie S, Johnson M, Abraham C, Barker D, Walker A, Group obotPT (2005). Making psychological theory useful for implementing evidence based practice: a consensus approach. Qual Saf Health Care.

[77] Grol R, Grimshaw J (2003). From best evidence to best practice: effective implementation of change in patients’ care. Lancet.

[78] Cane J, O’Connor D, Mitchie S (2012). Validation of the theoretical domains framework for use in behaviour change and implementation research. Implement Sci.

[79] Michie S, Fixsen D, Grimshaw J, Eccles M (2009). Specifying and reporting complex behaviour change interventions: the need for a scientific method. Implement Sci.

[80] Eccles M, Armstrong D, Baker R, Cleary K, Davies H, Davies S (2009). An implementation research agenda. Implement Sci.

