# Supplementary material for: Implementation of safety checklists in surgery: a realist synthesis of evidence
Source: Implement Sci. 2015 Sep 28;10:137. doi: 10.1186/s13012-015-0319-9 (PMC4587654; doi:10.1186/s13012-015-0319-9)
Supplement: Additional file 1: — Data synthesis: categories of information extracted from primary reports. [file 13012_2015_319_MOESM1_ESM.doc]

Additional file 1: Data synthesis: categories of information extracted from primary reports

| Contextual Factors (extracted from introduction, methods or findings sections) |
| --- |
| Participant characteristics (gender, race, age, experience, discipline, role) |
| Characteristics of setting (number ORs, type of surgeries, other) |
| Characteristics of the interventionist (number, discipline, other) |
| Characteristics of the intervention (theory, intended dose (number, length, frequency of contacts), intended duration, delivery mode, content, whether tailored to subgroups |
| Identification and/or analysis of moderators or mediators of the intervention’s effect if applicable |
| Authors’ speculations on effects characteristics of the intervention, participants, interveners, or setting had on implementation |
| Implementation Process (extracted from introduction, methods or findings sections) |
| Participants enrolled: number participated, number declined to participate |
| Participants’ level of interaction with implementation process |
| Did implementation involve the use of any of the following interventions?   1. Opinion leaders 2. Communication 3. Education sessions 4. Self-assessment 5. Clinical training 6. Audit and feedback 7. Environmental redesign 8. Rewards 9. Coercion 10. Performance data |
| Implementation approach   - Planned - Limited / none |
| Participants involved: actual dose (number, length, frequency), test of dose on intervention effects, perceptions of satisfaction or acceptability |
| Intervener/system delivery: findings from process evaluation or measures of fidelity/integrity, description of implementation process |
|  |
| Was there any evidence of tailoring of interventions to context? |
| Authors’ speculation on barriers and facilitators to successful implementation |
