# Supplementary material for: Implementation of safety checklists in surgery: a realist synthesis of evidence
Source: Implement Sci. 2015 Sep 28;10:137. doi: 10.1186/s13012-015-0319-9 (PMC4587654; doi:10.1186/s13012-015-0319-9)
Supplement: Additional file 2: — Feasibility/acceptability/fidelity studies of surgical checklist implementation interventions ( n = 35). [file 13012_2015_319_MOESM2_ESM.docx]

Additional File 2: Feasibility / acceptability / fidelity studies of surgical checklist implementation interventions (n=35)

| Author | Study Design | Organisational Context | Type of Checklist and Implementation Strategies | Reasons for Success or Failure | Checklist Fidelity and Reported Behavioural / Attitudinal Outcomes of Use |
| --- | --- | --- | --- | --- | --- |
| Askarian *et al.* 2015 [30] | Before and after using observations and audit | Iranian hospital | - 2008 WHO SSC - Checklist introduction supported by Iranian MOH - External group trained in SSC use - Educational packages - Staff presentations | • NR | - Obtaining information during timeout and sign out section reportedly improved after implementation |
| Bashford *et al.* 2014 [31] | Before and after using survey and chart audit | Ethiopian hospital | - 2008 SSC, adapted - Education and training - Dissemination of information - Feedback to staff - Staff evaluation using questionnaire | - Staged approach to implementation involving 4 steps - 5-month consultation period - Staff perceptions that improvements in compliance means that efforts to sustain are no longer required - Limited long term follow up and ongoing support - Lack of material resources beyond the implementation period (i.e., paper, pens) | - SSC compliance rates 83% 1 month after implementation, 65% after 8 months - Decrease in compliance rates of 20% over 12 month period after implementation - Sign-out most difficult to complete, and missed completely in 21% of cases |
| Bell & Pontin [57] | Descriptive | 2 UK Trusts | - 2008 WHO SSC, modified - Set up a Patient Safety Working Party in preparation for implementation - Checklist piloted in 1 of 2 hospitals prior to roll out at the other hospital | - Staff engagement necessary for implementation to be successful - Need to work with key individuals to identify issues or gaps in implementation | - Improvements in staff morale and communication reported by staff post checklist implementation |
| Berrisford *et al.* 2012 [58] | Prospective chart audit | UK Trust  959 patients undergoing thoracic surgery | - 2008 WHO SSC, adapted - Information about the SSC distributed through department directorates in surgery, anaesthetics and nursing - Human factors training - Monthly interdisciplinary meetings - Laminated A1 sized sheet used to guide checking process - Checklist lead by surgeon and anaesthetist while nurses listened in - Audit and feedback loop | - Routine / sustained use of time-out attributed to a combination of team who believed in the benefits of checklists, management support, simplicity of process, minimisation of documentation, proactive explanation to users and appropriate user feedback - Increase in safety culture | - Item compliance post implementation:   1. Sign-in checklist: pulse oximeter in place 97.2%, risk for > 500 mL blood loss: 97.9%  2. Time-out checklist, anaesthesia concerns 98.6%, essential images displayed 100%  3. VTE prophylaxis errors were identified in 53/959 (6%) of time-outs |
| Bittle 2011 [62] | Qualitative | NZ city hospital | - 2008 WHO SSC - Quality service improvement team coordinated implementation - Team meetings with coach | - Staff initially apprehensive but the checklist became an established practice - ‘Coaches’ from quality division assigned to roll out checklist - Feedback loop | - 1 near miss averted (incorrect surgery) - Reported incidents fell from 12-11 from the previous year |
| Bliss *et al.* 2012 [32] | Before and after using observations and audit | US tertiary referral hospital, 600 beds | - 2008 WHO SSC, adapted - 3 x 1 hour training sessions | - Some checklist items redundant (e.g., introductions) - Checklist activities engaged staff in a collegial framework | - Completion rates of sign-in 97.3%, timeout 98.6%, and sign-out 93.2% |
| Bohmer *et al.* 2012 [33] | Prospective controlled intervention study with surveys | German university hospital | - 2008 WHO SSC, modified - Implementation coordinated by researchers in Operative Medicine - Education sessions - Checklist introduced by department heads | - All specialties were involved in the adaption of the checklist to local context | - After checklist implementation, improvements noted in awareness of staff names and roles (p = .008), verification of consent (p < .0001), quality of inter-professional cooperation (p < .0001), patient related information such as risk factors, diagnosis etc. (p < .0001 to p =.046), |
| Calland *et al.* 2011 [47] | RCT, observations with and without checklist use (65 cases) | US teaching hospital | - Specific checklist developed for laparoscopic procedures - Education given to surgeons on how to use the specifically developed checklist | - Specifically developed checklist perceived as being more technically challenging - Performance of checklist dependent on team factors (i.e., personality, role, experience) | - Significant positive results for elements in the checklist cohort (p < .001)   1. Team introductions  2. Patient case presentation  3. Roles/responsibilities  4. Contingency planning |
| Conley *et al.* 2011 [60] | Qualitative  interviews | 5 US teaching hospitals | - 2008 WHO SSC - Support from hospital’s Vice President in Patient Safety - Rollout 2-6 months across hospitals - Local champions | - Implementation was incomplete at 3 hospitals - Implementation in 2 hospitals suspended because resistant culture or because they could not progress beyond pilot testing - Another hospital had less effective implementation because of a lack of strong leadership - Practice variation post rollout | - NR |
| Cullati *et al.* 2014 [59] | Descriptive study using  observations | Swiss university hospital, 38 ORs | - 2008 WHO SSC, adapted - Implementation strategies NR | - Designated roles had responsibility for each section on the checklist - Variation and inconsistency in timing of when each phase was conducted - Staff believed some SSC items were ambiguous - Hierarchical professional culture and lack of confidence | - Timeouts and sign-outs were conducted “quasi-systematically”, i.e., without using SCC as a visual reminder/reference - Compliance rates:   1. Timeout – 72% to 100%  2. Sign-out 19% to 86%   - 13% of Timeouts and 3% of Sign-outs were properly checked (all items validated) - Validation for complex procedures slightly increased with greater procedural risk - Surgeon was present in 96% Timeouts - Variation in individual item use and compliance |
| de Vries *et al.* 2009 [34] | Descriptive study using  observations  and interviews | Dutch university hospital | - SURPASS Checklist,   60 items   - Presentations about how to use checklist | - 34% interviewees reported lack of time to complete checklist - 66% interviewees forgot to use the checklist - 13% interviewees believed   that compliance would increase if consequences were attached to using the checklist   - 45% doctors interviewed suggested integrating the checklist into current hospital electronic information systems | - During 171 high risk surgeries, 593 process deviations were observed - Of those deviations covered on the checklist, 96% corresponded with an item on the checklist |
| Fourcade *et al.* 2012 [63] | Descriptive study using a random sample of 80 observed surgeries and interviews | 18 French oncology hospitals | - 2008 WHO SSC, modified - Implemented by National Federation of Cancer centres in collaboration with researchers | - Organisational and professional culture barriers identified - Perceived time constraints associated with checklist completion - Elements on the checklist perceived to be duplicated - Some items perceived as confusing as they were not part of routine practice - Poor communications between surgeons and anaesthetists - High staff turnover, new staff unfamiliar with SSC - Staff not actively engaged while performing checklist - Nurses concerned about the legal ramification of signing checklist - In 5/18 hospitals, boxes for checklist could be completed despite that the safety check were not performed | - Checklist performed in 90.2% of surgeries but only fully completed in 61.0% cases |
| Gillespie *et al.* 2010 [35] | Qualitative interviews | Australian hospital, 11 ORs | - 3-Cs safe surgery checklist – correct patient, correct site and correct procedure protocol, i.e., “timeout” - Endorsed by the Royal Australasian College of Surgeons | - Implementation of 3-Cs checklist left to senior nurses - Barriers to implementation included:   1. Haphazard implementation, responsibility devolved to senior nurses  2. Hierarchical team culture and silo mentality  3. Competing clinical priorities, with time constraints being identified as a primary barrier | - Compliance to the 3-Cs checklist was reported as being variable and inconsistent - Surgeons perceived by nursing staff as difficult to engage in use of 3-Cs checks |
| Haugen *et al.*  2013 [36] | Before and after using surveys | Norwegian hospital | - 2008 WHO SSC, modified - Randomised sequential roll out across surgical specialties - Implementation supported by Patient Safety Study Group of Bergan - Dissemination of information via emails, lectures, and videos - Change champions - Regular audit and feedback | - Use of team introductions may have increased cohesion, and thus influenced staffs’ perceptions - For checklist implementation to be effective, an organisation-wide culture change is critical - Implementation timeline may have been too short to obtain reported improvements in all safety culture domains | - Checklist compliance ranged from 77%-85% - Significant positive changes in safety culture relative to ‘frequency of events reported’ and ‘adequate staffing’ [20.25, 95% CI 20.47 to 20.07 and 0.21, 95% CI, 0.07–0.35], with higher scores in the intervention group |
| Haynes *et al* 2009*.* [37] | Before and after using observations and audit | Multinational studies across 8 countries | - 2008 WHO SSC - 2-step implementation plan - Local implementation teams at each hospital site - Introduction period from 1-4 weeks - Presentations, written information, recorded videos and guided education - Site visits by implementation team | - Checklist translated into the local language where appropriate - Modified to reflect the flow of care - Hospitals in low income and developing countries had limited resources (e.g., pulse oximeters, sterility indicators, antibiotics) which limited compliance to some checklist items | - Compliance across 6 safety indicators (airway, oximetry, IV lines, prophylactic antibiotics, verbal confirmation of patient’s identity and surgery site) increased from 34.2% to 56.7%, p <.0001 after implementation. - Adherence rates of team introductions, prebriefings and debriefings could not be measured |
| Helmio *et al.* 2011 [38] | Descriptive before and after study using surveys | ENT department in 4 Finnish hospitals | - 2008 WHO SSC - Information lectures x 3 before participating in pilot - Specific guidelines for use accessible and brief instructions on the back of the checklist | - Active leadership, regular audits and feedback | - Preoperative anaesthetic equipment checks increased from 71%-84% - Knowledge of OR members’ names and roles increased from 81%-94% - Successful communication 87%-96% - Discussing risks 38% |
| Kasatpibal *et al.* 2012 [18] | Descriptive  Survey | Thai university hospital  21,877 surgeries yearly | - 2008 WHO SSC, version NR - Circulating OR nurse participated in 2 meetings and 1-day data collection training session | - Low checklist compliance because surgical site marking materials unavailable, emergent procedures and Thai culture (i.e., do not put markings on the body) - Attitudes of surgeons resistant to use - Standards of practice to manage life-threatening issues already embedded into routine | - Compliance of various aspects of checklist high for life-threatening issues:   Verification of: patient name: 96.0%, incision site: 95.7%, procedure 95.9%   - 91% of patients confirmed identity, site, procedure and gave consent. Only 19% of surgical sites marked - Anaesthesia equipment and medication checked in 90% of cases. - Pulse oximeter applied in 95% of cases. - Allergies, difficulty airway, aspiration risk and risk of >500 mL blood loss assessed in 100% of cases |
| Kearns *et al.* 2012 [50] | Before and after using survey and direct observations | UK Trust  Obstetric ORs with 6,400 deliveries/year | - 2008 WHO SSC, version NR - Humorous posters - Before introducing the SSC, staff attitudes to safety surveyed | - All staff empowered to remind team members to perform checklist if forgotten - Success of the checklist related to a sense of ownership, allocation of responsibilities, and ongoing staff consultation | - Compliance with sign-in 61.2% after 3 months and 79.7% after 12 months - Compliance with sign out 67.6% after 3 months, and 84.7% after 12 months - 3 months after introduction, 50% (p = .026) staff felt familiar - 69.6% believed communication had improved - 30.4% (p = .025) believed in emergency cases the checklist was inconvenient - 75% patients asked reported that they noticed the checklist being performed while in OR - 93% of these patients reported feeling reassured that the checks were being done |
| Kwok *et al.* 2012 [39] | Before and after using chart audit | University hospital, Moldova, 600-700 surgeries per month | - 2008 WHO SSC, modified - Staged roll out over 1 month, adding 3 ORs per week - Local implementation team of surgeons, anaesthetists, nurses, hospital administrators - Coaching, education sessions - Formal meetings with implementers and staff | - Adherence increased with familiarity of use and experience | - Checklist used in 95% of cases - Completed in 90% of cases - Intraoperative indicators of communication improved 6 fold |
| Levy *et al.* 2012 [40] | Descriptive study using observations and surveys | US tertiary referral children’s hospital with 240 beds | - 2008 WHO SSC, modified - Posters and presentations - Physicians were not required to participate in all aspects of the education program - Fidelity of checklist use, unclear | - Inadequate education during implementation led to confusion about practical performance of checklist - Posters lacked practice instructions on how to perform the checklist - Checklist was not adapted for paediatric patients so may be less relevant | - SSC compliance reported at 100% on EMR - Only 4/172 cases completed more than 7/13 checkpoints - Reported confusion about timing and team member responsible for each section |
| Mainthia *et al.* 2012 [46] | Descriptive study using observations | US paediatric hospital | - Interactive electronic checklist of time-out checks - Introduction of whiteboards containing with checkboxes - After each check, the checklist items turn green - Once steps in time out process were complete, the text display changed from time out mode to case mode | - Active process of participation in checklist activity - Steps of time-out process verified contemporaneously rather than at once at the end | - Compliance with completion of checklist items post-implementation   1. 36.1% increase in time-out  2. Compliance of core items, pre-intervention: 49.7% ± 12.9%; Post-intervention at 1 month: 81.6% ± 11.4%  3. Post-intervention at 9 months: 85.8% ± 6.8%  4. Improvement in compliance with elements of  time out (p < .0001) |
| Norton & Rangel 2010 [45] | Descriptive | US paediatric hospital | - 2008 WHO SSC, modified - 3 x 5 foot posters in each OR - Launch included formal letter to all staff - Local champions from surgery, nursing and anaesthetics - Multiple training sessions - Dissemination of checklist use via hospital newsletter | - Use of paediatric checklist encouraged team communication - Allocated responsibility for each section to team members from nursing, anaesthetics and surgery | - In 80%-90% procedures, compliance with checklist - Staff perceived improvements in team communications - Checklist caught 1 near-miss during sign-in, several others during time-out, and 1 during sign-out |
| Pe’rez-Guisado 2012 [48] | Descriptive cross-sectional | Spanish hospital  1,684 surgeries | - 2008 WHO SSC, modified - Responsibility for sections of the checklist divided among surgeon, nurses and anaesthetists | - Local 10 question checklist already used, containing 8 items from the WHO SSC - Checklist compliance linked to hierarchical position | - Nurses achieved 99% implementation rates but surgeons and anaesthetists completed checklist in 79% and 72% respectively - Checklists fully completed in 39% of patients |
| Anonymous 2010 [41] | Descriptive | UK Trust with 8 ORs | - 2008 WHO SSC - Core group of patient safety experts developed strategies for implementation of SSC - Drop in educational sessions involving 120 staff - Piloted for 1 month in 2 hospitals in 62 surgeries - Staged roll-out | - A Trust-wide introduction - Importance of communicating with stakeholders beyond the core group - Adoption requires a culture change | - Within the first month of checklist introduction, usage rates increased from 33%-72% - Staff feedback was positive, most were keen to use the checklist - 1-month pilot identified 9 potential clinical incidents were avoided |
| Russ *et al.* [64] | Qualitative interviews,  119 interviews | UK, 10 hospitals | - 2008 WHO SSC, version NR | - Barriers and enablers of checklist implementation described in relation to team, checklist-specific, systems and organisational | NR |
| Rydenfalt *et al.*  [52] | Descriptive observational study  24 surgeries | Swedish hospital | - 2008 WHO SSC, adapted - Focussed on time-out checks - Implementation process NR | - Deviations in practice attributed to participants’ level of understanding of the intent of the checklist - Variations in perceived importance of individual checklist items and their relevance | - Staff introductions in 14/24 (58%) cases - 130/240 (54%) checklist items covered across 24 observed surgeries - Higher rates of compliance associated with patient ID, type of procedure and antibiotics - Lowest compliance associated with site of incision, OR nurse team reviews and imaging information - OR nurses did not participate in time-out |
| Sewel *et al.*2011 [53] | Before and after audits and surveys | UK teaching hospital, orthopaedic surgeries | - 2008 WHO SSC - 3 month pre-training prior to SSC introduction | - Initial introduction met with resistance as OR staff believed they already performed these checks - Increased infrastructure and an education program may improve staff perceptions of checklist use | - 77% OR staff believed SSC improved communication - 68% thought SSC improved patient safety - 80% want the checklist used if they had surgery |
| Sparkes & Rylah 2010 [42] | Descriptive  Chart audit | UK teaching hospital with  29 ORs | - 2008 WHO SSC, modified - 3 month pilot prior to roll out - Education support and training in use of SSC | - Despite agreement with the checklist in theory, there was resistance by senior staff - Checklist had to be signed by a team member, leading to fear of apportioning blame | - Post-implementation audit of 250 surgeries showed that team briefings occurred 77% of the time and timeouts occurred on 86% of occasions |
| Styer *et al.* 2011 [43] | Qualitative | US teaching hospital with 44 ORs | - 2008 WHO SSC, modified - Early endorsement by executive leadership - 2-weel trial with graduated introduction - Slide presentations and email updates | - Physician involvement essential for success - Controlled roll out - PDSA cycle used during implementation allowed for real time feedback - Each discipline should lead a section of the checklist - Standardisation of practice - Checklist adopted as hospital policy | - NR |
| Takala *et al.* 2011 [49] | Before and after using surveys | 4 Finnish university teaching hospitals | - 2008 WHO SSC, modified - 2-4 week implementation period - Nurses, anaesthetists and surgeons surveyed about OR practices, and repeated at 4-6 weeks after implementation | - NR | - Teams reported increased confirmation of patient identity and members’ names and roles (p< .001) - Surgeons reported increased discussion of critical event with anaesthetists (34.7%-46.2%, p <.001), and documented postoperative instructions |
| Truran *et al.* 2012 [51] | Before and after using audit | UK Hospitals | - 2008 WHO SSC, modified - 2 audits before and one 6 months after implementation | - NR | - Non-compliance with venous thromboembolism prophylaxis decreased after introduction of checklist from 6.9% to 2.1% |
| Vats *et al.* 2010 [54] | Descriptive  Chart audit | UK university hospital | - 2008 WHO SSC, modified - Clinical training | - Need a local champion as well as local leadership - Modified to context - Limited time given for training in use of checklist | - Notable improvements in safety processes such as antibiotic timing which increased from 57%-77% after the checklist was introduced |
| Vogts *et al.*2012 [55] | Before and after using direct observations | NZ city hospital | - 2008 WHO SSC, modified | - Low compliance rates attributed to a lack of linkage between a specific event in patient management, and the nurses tasked with these activities had competing priorities | - Compliance with ‘sign-in’ and ‘timeout’ sections decreased from 22.9% to 10% after checklist introduction - Compliance with ‘sign-out’ 2% |
| van Klei *et al.* 2012 [61] | Retrospective cohort  Chart audit | Dutch University hospital | - 2008 WHO SSC, modified - Implementation in accordance with Dutch Health Care Inspectorate - Regular information given to staff - Posters placed in all ORs | - Checklist completions less likely during emergency surgeries where patients have a higher risk of death. Raises questions for adjusting for patient acuity - Checklist completion devolved to nursing staff | - Checklist fully completed in 39% of all patients - Median number of items documented was 16/19 |
| Yuan *et al.* 2012 [44] | Before and after using observations and audit | 2 Libyan hospitals | - 2008 WHO SSC, modified - SSC implementation supported by Libyan MOH - 2-week training program consisting of lectures and guided learning - Local leaders | - Checklist fostered a shift culture on an individual and team level, to one that promoted patient safety - Successful in that the checklist expedited equipment procurement - Failure related to lack of consistent access to crucial resources and did not change hierarchical culture and team dynamics | - Overall improvement to checklist adherence ≥4/6 safety processes in one hospital (adjusted OR: 4.06; 95% CI: 2.18–7.57) but not the other (adjusted OR: 2.35, 95% CI: 0.82–6.73) |

*Abbreviations:* CI=Confidence interval*;* ENT=ear, nose and throat; SURPASS=SURgical PAtient Safety System, EMR= electronic medical record; UK=United Kingdom, US=United States, NR=not reported; MOH= Ministry of Health; PDSA= Plan, Do, Study, Act; WHO=World Health Organization
