# Supplementary material for: Implementation of safety checklists in surgery: a realist synthesis of evidence
Source: Implement Sci. 2015 Sep 28;10:137. doi: 10.1186/s13012-015-0319-9 (PMC4587654; doi:10.1186/s13012-015-0319-9)
Supplement: Additional file 3: — NPT analysis for evaluation of checklist implementation in surgery. [file 13012_2015_319_MOESM3_ESM.doc]

Additional file 3: NPT analysis for evaluation of checklist implementation in surgery

| NPT Components | Questions to consider within the NPT framework | Checklist implementation |
| --- | --- | --- |
| *Coherence* | Is the intervention easy to describe? | - Some end-users did not differentiate the intervention from current practice and were unable to perceive the proposed benefits to both surgical teams and patients. |
|  | Is it clearly distinct from other interventions? | - Clearly distinguishable from previous practice as the checks intended to be performed in a deliberate manner involving all team members. |
| (i.e., meaning and sense-making by participants) | Does it have a clear purpose? | - Perceptions of process duplication with checklist introduction. |
|  | Do end-users have a shared sense of its purpose? | - Checklist participation and item adherence appears dependent on the professional role and discipline. - Issues identified regarding the discipline lead of each phase of the checklist but role was often devolved to nurses. |
|  | What benefits will the intervention bring and to whom? | - Expected improvements in team cohesion and performance. - Potential minimisation of patient risk associated with specific checks. |
|  | Are these benefits likely to be valued by potential end-users? | - Some end-users were initially sceptical of the potential value of the checklist as a safety tool. - Perceptions that surgical teams were already doing these checks, albeit informally (i.e., not as a team). |
|  | Will it fit with the overall goals and activity of the organisation? | - Implementation of surgical safety checklists was considered an important strategy on which to build and improve the safety culture of the organisation. - Support for local leaders at the wider organisational level not always apparent, thus limiting their impact. |
| *Cognitive participation* | Are target user groups likely to think the intervention is a good idea? | - Concept of checklists in surgery supported in principle by expert professional groups. |
| (i.e., commitment and engagement by participants) | Will end-users see the point easily? | - Initial acceptance and embracement of checklists in practice was often dependent on end-users’ professional discipline, with some groups harder than others to convince. |
|  | Will they be prepared to invest time, energy and work in it? | - Implementation strategies (i.e., education, communication, local champions) curtailed by time and resource constraints. |
| *Collective action* | How will the intervention affect the work of user groups? | - In some contexts, end-users reported added workload as a result of checklist implementation. |
|  | Will it promote or impede their work? | - Time constraints sometimes impacted on checklist adherence especially in emergency surgeries or when lists were running over time. |
|  | Will staff require extensive training before they can use it? | - Some training required in its use in relation to timing, personnel, and significance of items. - Modification of work processes required, i.e., checks becomes a team-based activity rather than an individual activity. |
|  | How compatible is it with existing work practices? | - Some changes needed in current work processes to accommodate the addition of checklist use in practice. - End-users modified the checklist to reflect the flow of care. Despite these modifications, the level of item completion was variable. - Variability in processes use to enact the checklist, may be related to context. |
|  | What impact will it have on division of labour, resources, power, and responsibility between different professional groups? | - Level of participation was often based on discipline. - Nurses often led the checklist phases however had little power or authority to enact it. - Professional dissonance, checklist implementation had the potential to strain professional relations. |
|  | Will it fit with the overall goals and activity of the organisation? | - Notion of checklists to improve safety supported in most hospitals however organisational support was often nominal. - Professional leadership often determined success in implementation. |
| *Reflexive monitoring* | How are users likely to perceive the intervention once it has been in use for a while? | - Although participants perceived benefits, there was longitudinal decay in usage rates of checklist items and overall completion rates. |
| (i.e., participants reflect on or appraise the trial) | Is it likely to be perceived as advantageous for patients or staff? | - End-users reported work process improvements relative to team communication, safety culture and cooperation. - Patient satisfaction and reassurance that checks were being performed. |
|  | Will it be clear what effects the intervention has had? | - Audit and feedback given to end-users in relation to checklist compliance and performance monitoring (i.e., number of near misses averted, VTE adherence rates). |
|  | Can staff contribute feedback about the intervention once it is in use? | - Reflection on the ongoing use of implementation strategies seemed limited. - Decay in consistent use of checklist over time suggests periodic review and ongoing adaption was limited. |
|  | Can the intervention be adapted / improved on the basis of experience? | - Initial modifications to context and work flow patterns however, ongoing adaptations not reported. - Tailoring of the checklist implementation occurred rarely, if at all. |
