# Supplementary material for: Implementation of safety checklists in surgery: a realist synthesis of evidence
Source: Implement Sci. 2015 Sep 28;10:137. doi: 10.1186/s13012-015-0319-9 (PMC4587654; doi:10.1186/s13012-015-0319-9)
Supplement: Additional file 4: — CMO configuration in relation to sustainability of checklists in surgery. [file 13012_2015_319_MOESM4_ESM.doc]

Additional file 4: CMO Configuration in relation to sustainability of checklists in surgery

| **Context *(C)*** | ***+*** | **Mechanism *(M)*** | ***→*** | **Outcome *(O)*** |
| --- | --- | --- | --- | --- |
| - Need to improve patient safety and outcomes in surgery - Culture of surgery - Professional identification - Organisational and departmental factors - Workload and time pressures - Characteristics of the implementers |  | - Active leadership - Support strategies - Process simplification - Reflexivity |  | - Improved interdisciplinary communication - Improved clinical processes - Improved safety culture - Improved item compliance rates - Enhanced staff morale |
|  |  |
|  |  |
|  |  |
|  |  |
|  |  |
